# Effective Combination of the Metal Centers in MOF-Based Materials toward Sustainable Oxidation Catalysts

**DOI:** 10.3390/ma16083133

**Published:** 2023-04-16

**Authors:** Alexandre M. Viana, Francisca Leonardes, Marta C. Corvo, Salete S. Balula, Luís Cunha-Silva

**Affiliations:** 1 LAQV-REQUIMTE, Department of Chemistry and Biochemistry, Faculty of Sciences, University of Porto, 4169-007 Porto, Portugal; up201405091@edu.fc.up.pt (A.M.V.); leonardes.francisca@gmail.com (F.L.); 2 CENIMAT/I3N, Departamento de Ciência dos Materiais, Faculdade de Ciências e Tecnologia, Universidade Nova de Lisboa, 2829-516 Caparica, Portugal

**Keywords:** zeolitic imidazolate frameworks, polyoxometalates, nanocomposite material, oxidative desulfurization, heterogeneous catalysis

## Abstract

A successful encapsulation of Keggin-type polyoxomolybdate (H_3_[PMo_12_O_40_], PMo_12_) into metal-organic framework (MOF) materials with an identical framework but distinct metal centers (ZIF-8 with Zn^2+^ and ZIF-67 with Co^2+^) was accomplished by a straightforward room-temperature procedure. The presence of Zn^2+^ in the composite material PMo_12_@ZIF-8 instead of Co^2+^ in PMo_12_@ZIF-67 caused a remarkable increase in the catalytic activity that achieved a total oxidative desulfurization of a multicomponent model diesel under moderate and friendly conditions (oxidant: H_2_O_2_ and solvent: ionic liquid, IL). Interestingly, the parent ZIF-8-based composite with the Keggin-type polyoxotungstate (H_3_[PW_12_O_40_], PW_12_), PW_12_@ZIF-8, did not show the relevant catalytic activity. The ZIF-type supports present an appropriate framework to accommodate active polyoxometalates (POMs) into their cavities without leaching, but the nature of the metallic center from the POM and the metal present in the ZIF framework were vital for the catalytic performance of the composite materials.

## 1. Introduction

Fossil fuels continue to be the major energy source that is applied for several purposes on which we are reliant, such as transportation; thus, much attention is now being focused on fossil fuel consumption as well as its production and processing, answering the calls for the ever-growing need for sustainable development [1]. One of the most relevant problems associated with the combustion of oil fuels is the emission of sulfur-derived products to the atmosphere, such as various sulfur oxides and particulate metal sulfates, which stem from the different sulfur-containing compounds (SCCs) that make up part of the original composition of crude oil, including thiophenes, sulfides and disulfides, mercaptans, dibenzothiophenes, and many other derived species [2]. If these SCCs are not discarded from the fuel matrix before combustion, their emissions will cause some environmental issues that are linked to acid rain and associated with several public health problems [3]. To mitigate these serious problems, the petrochemical industry must abide by international legislative regulation concerning the sulfur content that is present in processed fossil fuels, resorting to desulfurization processes [4]. The presence of these compounds is also undesirable during some stages of the refining process, as they can promote equipment corrosion and deactivation of the catalysts. Hydrodesulfurization (HDS) is the main approach that is currently used to remove SCCs from fuels in industrial plants, as it manages to efficiently convert the sulfur content to hydrogen sulfide and sulfur-free organic compounds [5]. HDS is dependent on high temperatures and pressures for hydrogen-consuming catalytic processes, which, knowing its lower efficiency to eliminate heterocyclic compounds such as thiophenes, makes it an undesirably costly method to produce sulfur-free fuels [6].

Oxidative desulfurization (ODS) is an emerging and potentially cost-effective approach to desulfurize fuel derivatives, since it can accomplish the efficient removal of the most refractory thiophenes and dibenzothiophenes under sustainable conditions, i.e., low pressure and temperature, and avoid the consumption of hydrogen [7]. The ODS process is performed in two main steps: initially, the oxidation of SCCs to their corresponding sulfones or sulfoxides occurs, in which an oxidizing agent is needed for the contribution of one or two oxygen atoms, respectively; after, the extraction occurs, for which a suitable extracting solvent needs to be selected. Naturally, the first stage is enabled by catalytic systems, for which an adequate catalyst must be appointed, and many different types of materials can be considered for this task; polyoxometalates (POMs) are credited as one of the most interesting ones [8].

POMs are polyatomic ions based on transition metal oxyanions that are recognized for their structural diversity, interesting chemical properties, and potential applicability in several areas. These are assembled by MO_x_ coordination polyhedra (M: transition metal; x: 4, 5 or 6), which can result in various topological motifs [9]. Keggin arrangements are well-known POM structures that share a [X^n+^M_12_O_40_]^(8−n)−^ (X stands for a heteroatom form block p or d) formula that is built around a central XO_4_ tetrahedron that is enclosed in twelve MO_6_ octahedrons and organized in groups of M_3_O_13_ units that are linked by O vertex atoms to form a spherical structure with tetrahedral symmetry. Usually, POMs are effective catalysts in a vast number of distinct reactions, and the Keggin POMs have revealed themselves to be particularly useful as selective catalysts of oxidation reactions, where ODS reactions can be included [10,11]. Still, these can be associated with the usual drawbacks that are related to homogeneous catalysts, namely, poor recyclability potential, which is a very important parameter to consider when looking for an efficient oxidative desulfurization catalytic system as an alternative for the HDS process. Many strategies have been proposed to solve this problem, such as their heterogenization by encapsulation in suitable support materials, such as porous metal–organic frameworks (MOFs) [12]. This new family of materials includes coordination polymers formed by metal cations or clusters interconnected by organic linkers (ligands) originating from crystalline and microporous coordination frameworks. MOFs are very popular due to their structural diversity and their simply tunable properties, giving them enormous potential for a wide range of applications, such as gas separation and/or storage, chemical sensors, heterogeneous catalysts, platforms for drug delivery, and energy storage [13,14,15,16,17].

**Figure 1:**
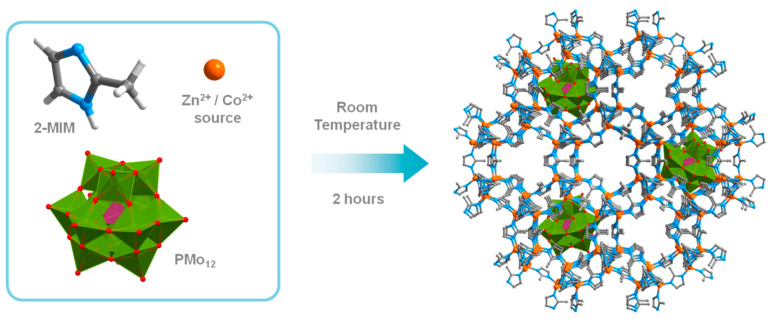
Schematic representation of the in situ sustainable preparation of the PMo_12_@ZIF composite materials.

Following our research endeavors on functional crystalline materials and the oxidative desulfurization process [18,19,20,21,22,23,24], also including zeolitic imidazolate framework (ZIF) materials [25,26], an innovative study that compares the catalytic efficiency of different combinations of polyoxometalates encapsulated in ZIF supports with distinct metallic centers is reported. ZIFs are a well-known subclass of crystalline and porous MOFs that have frameworks analogous to those found in zeolites. The encapsulation of active Keggin POMs as H_3_[PMo_12_O_40_] (PMo_12_) and H_3_[PW_12_O_40_] (PW_12_) was performed in situ with MOF assembling (Figure 1). ZIF-8 and ZIF-67 are highly stable isostructural M(2-mim)_2_ frameworks (M: respectively Zn^2+^ and Co^2+^; 2-mim: 2-methylimidazole) with sodalite-type topology and cavities built upon MN_4_ tetrahedral units [27]. The key reason for the selection of these two structures as hosts for guest POMs was the match between their dimension (10 Å) and the MOFs pore size (11.6 Å), which was related to the smaller pore window (3.4 Å) and could prevent leaching [28]. Therefore, probably only one POM unit can fit into a cage of ZIF materials. The small pore window will guarantee the absence of POM leaching but can simultaneously hinder the diffusion of the reactants to meet the active center that is entrapped in the ZIF cage. This is probably the reason for the scarce amount of work reported in the literature that uses ZIF materials as heterogeneous catalysts in the liquid phase [25,26,29,30].

## 2. Experimental Section

### 2.1. Materials and Characterization Methods

Phosphomolybdic acid (H_3_PMo_12_O_40_.xH_2_O, PMo_12,_ microscopy grade; Sigma-Aldrich, Waltham, MA, USA), acid phosphotungstic (H_3_PW_12_O_40_.xH_2_O, PW_12,_ microscopy grade, Sigma-Aldrich), cobalt(II) nitrate (Co(NO_3_)_2_.6H_2_O, > 99.8%, Sigma-Aldrich), Zinc(II) nitrate (Zn(NO_3_)_2_.4H_2_O > 98.5%, Merck, Rahway, NJ, USA), 2-methylimidazole (C_4_H_6_N_2_, 99.0%, 2-mIm, Sigma-Aldrich), 1-butyl-3-methylimidazole hexafluorophosphate (C_8_H_15_F_6_N_2_P > 97%, bmImPF_6,_ Sigma-Aldrich), tetradecane (C_14_H_30_ > 99,0%, Sigma-Aldrich), hydrogen peroxide (H_2_O_2_, aqueous 30%, Sigma-Aldrich), *N,N-*dimethylformamide (C_3_H_7_NO, DMF, 99,99%, Fisher, Waltham, MA, USA), ethanol (C_2_H_5_OH, EtOH > 99.8%, Fisher), methanol (CH_3_OH, MeOH, analytical reagent grade, Fisher), acetonitrile (CH_3_CN, MeCN > 99.5%, Fluka, Buchs, Switzerland), benzothiophene (C_8_H_6_S, BT, > 95%, Fluka), *N*-octane (C_8_H_18_ > 99.0%, Acros Organic, Geel, Belgium), dibenzothiophene (C_12_H_8_S, 98%, DBT, Sigma-Aldrich), and 4-methyldibenzothiophene (C_13_H_10_S, MDBT, 96%, Sigma-Aldrich) 4,6-dimethyldibenzothiophene (C_14_H_12_S, DMDBT, 95%, Acros Organic) were acquired from commercial sources and used as received.

Fourier-transformed infrared (FTIR) spectra were acquired in the attenuated total reflectance (ATR) operation mode of a PerkinElmer (Waltham, MA, USA) FTIR System Spectrum BX spectrometer, and all the representations are shown in arbitrary units of transmittance. Powder X-ray diffraction (XRD) patterns were obtained at room temperature on a Rigaku (Tokyo, Japan) Geigerflex diffractometer operating with a Cu radiation source (λ_1_ = 1.540598 Å; λ_2_ = 1.544426 Å; λ_1_/λ_2_ = 0.500) and in a Bragg–Brentano *θ*/2*θ* configuration (45 kV, 40 mA). Intensity data were collected by a step-counting method (step 0.026°) in continuous mode in the 3 ≤ 2*θ* ≤ 50° range, and all the representations are shown in arbitrary units of intensity. Scanning electron microscopy (SEM) and electron dispersive X-ray spectroscopy (EDS) analysis were performed in a FEI (Lausanne, Switzerland) Quanta 400 FEG ESEM high-resolution scanning electron microscope equipped with an EDAX Genesis X4M spectrometer working at 15 kV. Samples were coated with an Au/Pd thin film by sputtering using a SPI Module Sputter Coater equipment. Solid state ^31^P nuclear magnetic resonance (NMR) spectra were acquired with an 11 T (500 MHz) AVANCE II+ Bruker spectrometer operating at 202.45 MHz, equipped with a BBO probe head. The samples were spun at the magic angle (MAS) at a frequency of 5 kHz in 4 mm-diameter rotors at room temperature and the spectra were obtained with proton cross-polarization (CPMAS) with a contact time of 5.0 ms and the recycle delay was 5.0 s. Inductively coupled plasma optical emission spectroscopy (ICP-OES) was used to quantify Mo concentrations in various samples, resorting to a PerkinElmer Otima 4300 DV. N_2_ adsorption–desorption isotherms were collected at −196 °C with a gas porosimeter Micromeritics ASAP 2010. Pre-outgassing of the analyzed samples was carried out at 150 °C for 2 h. Catalytic reactions were periodically monitored by GC-FID analysis carried out in a Bruker 430-GC-FID chromatograph. Hydrogen was used as carrier gas (55 cm.s^−1^) and fused silica Supelco (St. Louis, MO, USA) capillary columns SPB-5 (30 m × 0.25 mm i. d.; 25 μm film thickness) were used.

### 2.2. Materials Preparation

Supports ZIF-8 and ZIF-67

The support materials were prepared by an adaptation of the experimental procedure previously reported [28]. Briefly, primary solutions of Zn(NO_3_)_2_.4H_2_O or Co(NO_3_)_2_.6H_2_O (2.5 mmol) in MeOH (25 mL) were stirred magnetically for 15 min to prepare the Zn-based MOF (ZIF-8) and Co-based MOF (ZIF-67), respectively. After, solutions of 2-mIm (19.8 mmol) in MeOH (25 mL) were prepared and slowly added to the respective metal-containing solutions. The Zn- and Co-based resulting reactional mixtures were stirred (magnetically) for 150 min, at room temperature and ambient pressure. Lastly, the obtained solid materials were isolated by centrifugation, washed with MeOH (five times), and dried under vacuum at 60 °C for 12 h.

PMo_12_@ZIF-8

This procedure is adaptation of the previously mentioned methods using a one-pot procedure for the preparation of POM composites. An aqueous solution (10 mL) of PMo_12_ (125 mg) was joined to the primary Zn^2+^ solution: 2.5 mmol of Zn(NO_3_)_2_.4H_2_O in 25 mL of MeOH. This resulting mixture was stirred (magnetically) for 30 min, after which 2-mIm methanolic solution (19.8 mmol in 25 mL of MeOH) was likewise added. The resulting reactional mixtures were stirred (magnetically) for 150 min at room temperature and ambient pressure. Lastly, the obtained solid materials were isolated by centrifugation, washed with MeOH (five times), and dried at under vacuum at 60 °C for 12 h. Atomic ratio of Zn/Mo = 1.86.

PW_12_@ZIF-8

An aqueous solution (10 mL) of PW_12_ (125 mg) was joined to a Zn^2+^ solution: 2.5 mmol of Zn(NO_3_)_2_.4H_2_O in 25 mL of MeOH. This resulting mixture was stirred (magnetically) for 30 min, after which 2-mIm methanolic solution (19.8 mmol in 25 mL of MeOH) was likewise added. The resulting reactional mixtures were stirred (magnetically) for 150 min at room temperature and ambient pressure. Lastly, the obtained solid materials were isolated by centrifugation, washed with MeOH (five times), and dried at under vacuum at 60 °C for 12 h. Atomic ratio of Zn/W = 1.41.

PMo_12_@ZIF-67

An aqueous solution (10 mL) of PMo_12_ (125 mg) was joined to the initial Co^2+^ solution: 2.5 mmol of Co(NO_3_)_2_.6H_2_O in 25 mL of MeOH. This resulting mixture was stirred for 30 min, after which 2-mIm methanolic solution (19.8 mmol in 25 mL of MeOH) was added. The resulting reactional mixtures were stirred for 150 min at room temperature and ambient pressure. Lastly, the obtained solid materials were isolated by centrifugation, washed with MeOH (five times), and dried at under vacuum at 60 °C for 12 h. Atomic ratio of Co/Mo = 0.67.

### 2.3. Oxidative Desulfurization Studies

The prepared ZIF supports and the POM@ZIF composites were evaluated as heterogeneous catalysts for ODS systems in a multicomponent model diesel comprising the SCCs, BT (500 ppm od S), DBT (500 ppm of S), MDBT (500 ppm of S), and DMDBT (500 ppm of S) in *n*-octane. The reactions were performed in a closed vessel, under air in a closed borosilicate vessel with a magnetic stirrer and immersed in a thermostatically controlled liquid paraffin bath at 70 °C. Oxidative catalytic desulfurization experiments were performed in a biphasic system composed by the model diesel and [BMIM]PF_6_ as extraction solvent. In a representative experiment, a certain amount of the catalyst material containing an equivalent of 3 mmol of active center POM was added to 0.75 mL of [BMIM]PF_6_ and 0.75 mL of model diesel, and this mixture was stirred for 10 min at 70 °C. The oxidative catalytic step was initiated with the addition of aqueous H_2_O_2_ 30% (75 μL) to system. Tetradecane was used as a standard in the periodical monitorization of the sulfur content by GC analysis. At the end of each reaction, catalysts were recovered by centrifugation, washed carefully, and for three times with MeCN and EtOH, they were dried at 60 °C overnight under vacuum and reused in the subsequent catalytic cycle, which had identical reaction conditions. The desulfurization was quantified by analyzing the model diesel phase, which was withdrawn from upper phase (20 μL) and analyzed with addition of tetradecane as standard by gas chromatography to quantify the total sulfur content. Finally, the desulfurization efficiency (%) was calculated according to the following formula: Desulfurization efficiency (%) = (initial sulfur content-residual sulfur content)/(initial sulfur content) ×100 %.

## 3. Results and Discussion

### 3.1. Materials Characterization

**Figure 2:**
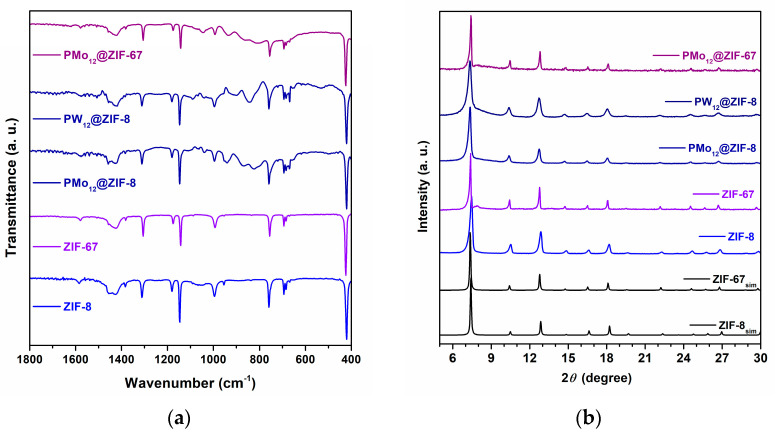
FTIR spectra (**a**) and Powder XRD patterns (**b**) obtained for ZIF-8, ZIF-67, and POM@ZIF composite materials (PMo_12_@ZIF-8, PW_12_@ZIF-8, and PMo_12_@ZIF-67).

The supporting materials ZIF-8 and ZIF-67 and the composites PMo_12_@ZIF-8, PW_12_@ZIF-8, and PMo_12_@ZIF-67 were prepared through a straightforward room-temperature experimental procedure based on the previously reported synthesis methods [31,32]. All the isolated materials were characterized by powder XRD, FTIR spectroscopy, SEM/EDS, and N_2_ adsorption–desorption isotherms. The vibrational spectra of the materials depict that the main absorption bands are predictable from the MOF structures (Figure 2a): a very strong band observed around 420 cm^−1^ assigned to metal-nitrogen coordination, strong and weak bands from 600 up to 1500 cm^−1^ can be attributed to C–N ring bonds, and a weak vibration band seen at 1580 cm^−1^ is assigned to the C=N bond [33,34]. Additionally, in the composite materials (PMo_12_@ZIF-8, PW_12_@ZIF-8, and PMo_12_@ZIF-67) spectra, a group of medium intensity bands are evident in the interval from 770 to 975 cm^−1^. These absorption bands can be assigned to Mo=O and Mo–O, or W=O and W–O, bond vibration bands from PMo_12_ or PW_12_ incorporated in the ZIFs vacancies, respectively [35]. Weak protuberances between 1000 and 1100 cm^−1^ can be associated with P–O bond vibrations and further support these results.

The powder XRD patterns registered for the pristine MOF samples ZIF-8 and ZIF-67 (Figure 2b) are consonant with the previously reported diffraction patterns for these crystalline structures, revealing the expected characteristic peaks with the correct relative intensities [36]. Furthermore, the PMo_12_@ZIF-8, PW_12_@ZIF-8, and PMo_12_@ZIF-67 samples revealed comparable unique-phase diffraction patterns, suggesting the effective formation of ZIF structures as composite frameworks. The absence of characteristic reflections of the POMs points to the random distribution of POMs in the framework of the ZIFs, most probably as a consequence of an effective and nonregular impregnation of the POMs in the cavities of the ZIFs.

**Figure 3:**
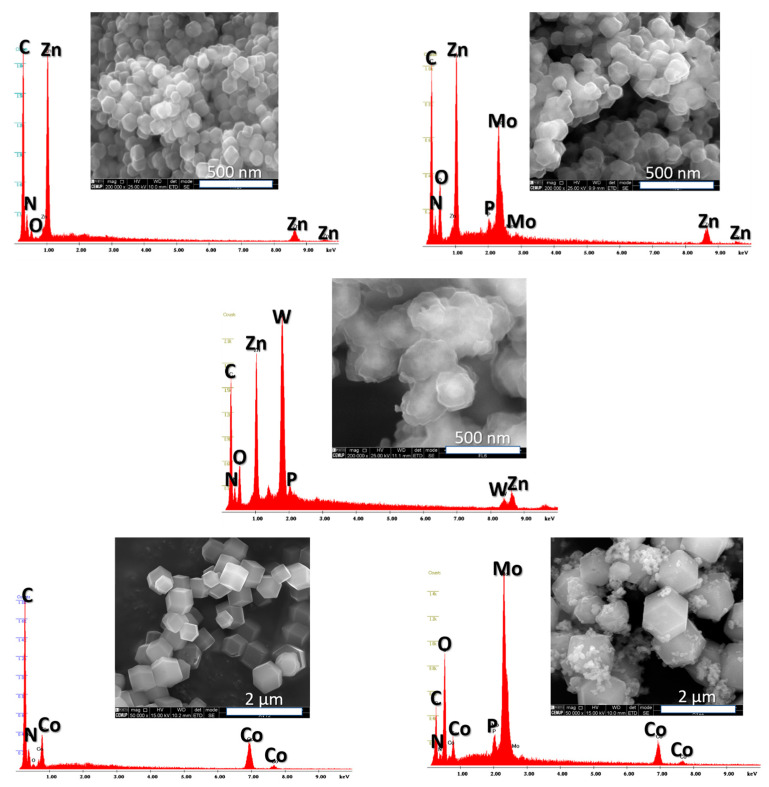
SEM micrographs and corresponding EDS spectra recorded for ZIF-8 (**above**, **left**), PMo_12_@ZIF-8 (**above**, **right**), PW_12_@ZIF-8 (**middle**), ZIF-67 (**below**, **left**), and PMo_12_@ZIF-67 (**below**, **right**).

SEM micrographs obtained for ZIF-8 and ZIF-67 samples reveal an aggregation of uniform particles with a rhombic dodecahedron morphology and regular sizes distributed around 100 nm and between 600 and 800 nm, respectively (Figure 3). Interestingly, the PMo_12_@ZIF-8 micrography shows particles with an identical size but with a lower degree of morphological uniformity (Figure 3 above, right). In these, the regular distribution of Mo and P revealed by the EDS analysis suggests a uniform distribution of the PMo_12_ on ZIF-8. Particles with an irregular size distribution are evidenced for PMo_12_@ZIF-67 (Figure 3 below, right). The EDS spectrum recorded for this sample shows Mo and P peaks, although distributed in an irregular fashion through the sample, as smaller particles that are associated with a higher Mo content (Figure 3 below, right).

**Figure 4:**
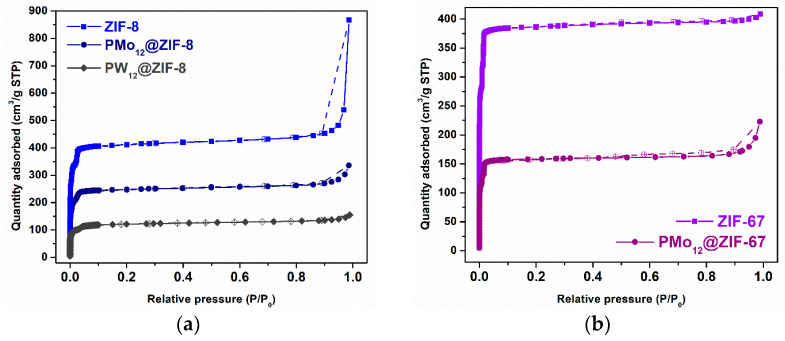
N_2_ adsorption–desorption isotherms at −196 °C of both MOFs and POM@MOF composite materials: (**a**) ZIF-8 and ZIF-8 based materials; (**b**) ZIF-67 and ZIF-67 based materials. Filled and unfilled symbols relate to adsorption and desorption processes, respectively.

N_2_ sorption isotherms of both MOFs and derived POM@MOF composite materials were acquired (Figure 4), and the estimated BET parameters are systematized in Table 1. All analyzed samples show type-I isotherms that are related to their expected microporosity. The specific surface areas calculated for ZIF-8 and ZIF-67 are in accordance with previously reported values for these structures [31,37]. An introduction of the POM units inside the MOFs cavities should result in a lower available surface area for N_2_ absorption. The consistently lower surface areas calculated for POM@MOFs are evidence of the successful preparation of these materials. PMo_12_@ZIF-8 and PW_12_@ZIF-8 were studied by ICP-OES to determine Mo or W concentrations and quantify PMo_12_ (0.15 mmol/g) or PW_12_ (1.6 mmol/g) inserted in the cavities of MOFs, in order to calculate the catalyst quantities that correspond to 3 μmol of an active POM to be used in ODS.

### 3.2. Catalytic Desulfurization Studies

Oxidative desulfurization studies were carried out at 70 °C in a biphasic system based on equal volumes of the model diesel and extraction solvent. The ionic liquid 1-butyl-3-methylimidazole hexafluorophosphate ([BMIM]PF_6_) was utilized as an extraction solvent and the oxidizing agent used was the aqueous hydrogen peroxide. The model diesel was prepared with various SCCs representative of the most refractory sulfur content present in diesel fuels, namely BT, DBT, MDBT, and DMDBT. Initially, an extractive desulfurization occurred every 10 min by mixing both liquid phases (1:1) [BMIM]PF_6_/model diesel. After this, the equilibrium of sulfur components extracted from diesel in the ionic liquid phase was reached, and to increase the desulfurization efficiency, the oxidant H_2_O_2_ is added to the system to cause the oxidative desulfurization. When the sulfur components that are present in the extraction phase are oxidized to sulfones and/or sulfoxides, more sulfur compounds are extracted from the diesel phase (ECODS, extraction, and catalytic oxidative desulfurization system).

**Figure 5:**
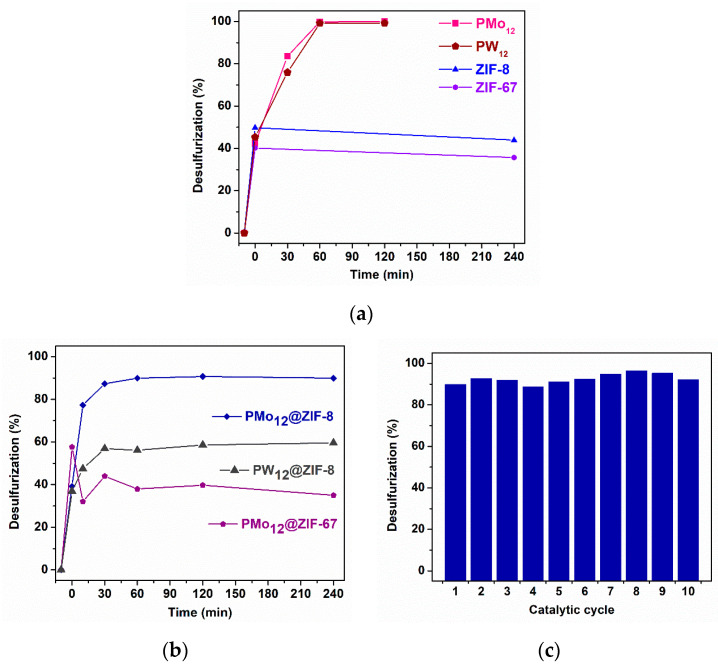
(**a**) Catalytic behavior of homogeneous POMs, PMo_12_ and PW_12_, and supporting MOFs, ZIF-8 and ZIF-67. Assays were performed at 70 °C in a model fuel/[BMIM]PF_6_ biphasic system with 3 μmol of POM or 15 mg of MOF. ODS reaction starts at 0 min with the addition of aq. H_2_O_2_, after a 10 min extraction step. (**b**) Desulfurization profile of a multicomponent model fuel using PMo_12_@ZIF-8, PW_12_@ZIF-8, and PMo_12_@ZIF-67 catalysts, [BMIM]PF_6_ extraction solvent, H_2_O_2_ oxidant, at 70 °C. (**c**) Recycling desulfurization process for ten consecutive cycles (2 h each reaction) using PMo_12_@ZIF-8 catalyst.

Preliminary ECODS tests were performed using the homogeneous catalytic active centers PMo_12_ and PW_12_, and also the support materials ZIF-8 and ZIF-67, to assess their inherent catalytic behavior (Figure 5a). PMo_12_ and PW_12_ promoted near total desulfurization of the model diesel (99.8%) after 1 h of reaction time, while both ZIF-8 and ZIF-67 did not exhibit any catalytic activity toward ODS. Further, the composite materials PMo_12_@ZIF-8, PMo_12_@ZIF-67, and PW_12_@ZIF-8 were evaluated as heterogeneous catalysts for ECODS processes (Figure 5b), with the POMs as the active center since the ZIF supports did not show any catalytic activity. As the parent MOF, PMo_12_@ZIF-67 does not show any catalytic activity, which suggests that the active PMo_12_ incorporated in the ZIF-67 framework cannot achieve its role as a catalyst. On the other hand, PMo_12_@ZIF-8 displays heterogeneous catalytic behavior toward ECODS, as it promotes high desulfurization of the model diesel (90.7%) after 2 h. At the end of this first catalytic cycle, the material was recovered, washed repeatedly, and dried. After this, the PMo_12_@ZIF-8 was used in nine consecutive ECODS cycles for 2 h. The guaranteed, consistent catalytic results confirm a great recycling potential (Figure 5b). Additionally, comparing the catalytic performances of PW_12_ and PMo_12_ incorporated into the ZIF-8 framework, it is possible to observe that the composite PW_12_@ZIF-8 presents much lower oxidative desulfurization efficiency, since the desulfurization only increased by 20% after the initial extraction step, instead of the 50% increase obtained using the PMo_12_@ZIF-8 catalyst. This low activity of PW_12_@ZIF-8 must be due to the low accessibility of the PW_12_ active center for interaction with sulfur compounds and oxidants, which is probably caused by its higher POM loading (approximately ten times more) than the PMo_12_@ZIF-8 composite. On the other hand, the PMo_12_@ZIF-67 composite presents a PMo_12_ loading that is only slightly higher than the PMo_12_@ZIF-8 composite; however, an absence of oxidative catalytic activity was found when using the ZIF-67 composite.

A comparative study was performed between the active PMo_12_@ZIF-8 and the other reported PMo_12_-based MOF catalysts used for the oxidative desulfurization of refractory model fuels. The results are summarized in Table 2. It is possible to observe that the active PMo_12_ active center was immobilized in several MOF structures, including UiO-66, UiO-67, and ZIF-6; however, most of these PMo_12_@MOFs catalysts were tested in single model fuels mainly containing the refractory sulfur compounds that are the easiest to oxidize and remove from model and real fuels, the DBT [38,39,40,41,42,43]. An identical multicomponent model diesel was used by Granadeiro et al. by incorporating PMo_12_ into porous NH_2_-MIL-101(Cr) [21]. Using this composite, near-complete desulfurization was achieved after 2 h; however, the recycling capacity was only verified up to three consecutive cycles. Last year, Zhou et al. used the PMo_12_@MOF-199 catalyst to desulfurize a 4,6-DMDBT single model fuel under aerobic reaction conditions [44]. Using molecular oxygen as an oxidant, a higher reaction temperature of 120 °C was needed to achieve 90% desulfurization after a short reaction time [44]. This was a successful result since 4,6-DMDBT is one of the most stubborn sulfides in fuel oil. Further, the PMo_12_@MOF-199 catalyst showed itself to be recyclable and stable under aerobic conditions. Yang et al. presented a porous ionic liquid catalyst using ZIF-8 support [42]. The intention to use the PIL functional group promoted the contact between the oxidant H_2_O_2_ and the active center PMo_12_. However, only single model DBT fuel was used, and even if complete desulfurization was achieved, a longer period of time (2 h) was needed without information concerning the stability and recycling capacity of this catalyst [42]. In 2021, Fernandes et al. used a model diesel containing three different sulfur compounds (1-BT, DBT, and 4-MDBT) that were completely desulfurized after 1 h of using the PMo_12_@MOF-808 catalyst. Stability and recycle capacity were confirmed [45]. This last composite is a promising one with a high efficiency and stability; however, its activity to desulfurize the hardest sulfur compound to be oxidized and extracted (4,6-DMDBT) is unknown.

**Figure 6:**
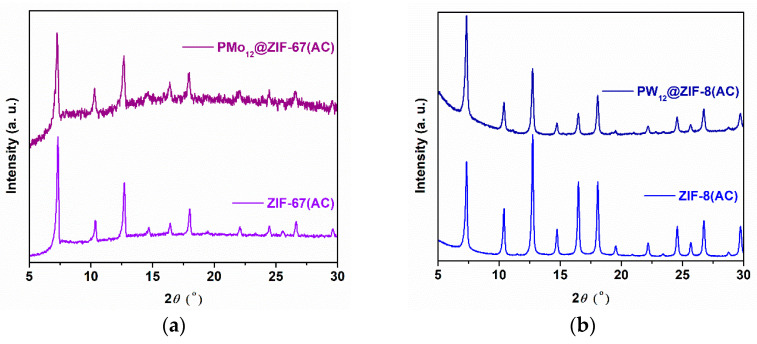

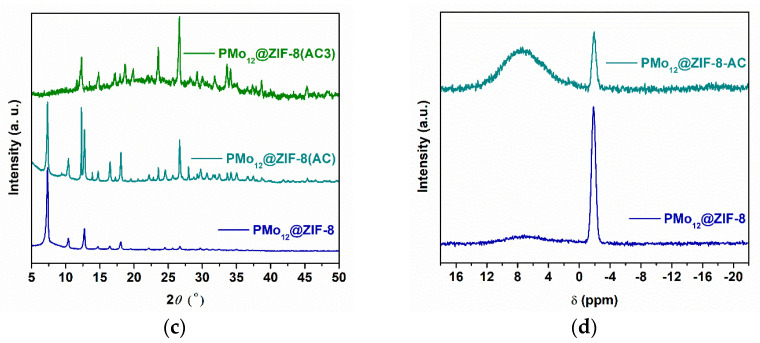
Powder XRD patterns obtained for recovered materials after catalysis (AC): (**a**) ZIF-67(AC) and PMo_12_@ZIF-67(AC); (**b**) ZIF-8(AC) and PW_12_@ZIF-8(AC); (**c**) PMo_12_@ZIF-8, PMo_12_@ZIF-8(AC) and PMo_12_@ZIF-8(AC3) (AC3 meaning after the 3rd catalytic cycle). (**d**) ^31^P MAS NMR spectra obtained for PMo_12_@ZIF-8 and PMo_12_@ZIF-8-AC.

The pristine support and the composite materials were recovered, washed, and dried after catalytic experiments (AC), and characterized to assess the distinguishing structural features after catalytic use. The powder XRD patterns obtained for ZIF-67(AC) and PMo_12_@ZIF-67(AC) (Figure 6a) correspond to the original diffractograms and show structural maintenance for both samples. The same is found for ZIF-8(AC) and for PW_12_@ZIF-8(AC) (Figure 6b): even though the registered relative peak intensity slightly varies, the crystalline structure of ZIF-8 seems stable under catalytic conditions. Active PMo_12_@ZIF-8 apparently undergoes structural transformations that are manifested after the third catalytic reaction, based on inconsistencies between the obtained diffractogram and the one recorded for ZIF-8 and PMo_12_@ZIF-8 without catalytic use (Figure 6c). These structural changes may explain the distinct catalytic behavior observed for PMo_12_@ZIF-8 and PMo_12_@ZIF-67 composites, since immobilized PMo_12_ was expected to be comparably accessible for catalysis in both structures. To further investigate the stability of the active center Pmo_12_, an analysis of the ^31^P MAS NMR spectroscopy of PMo_12_@ZIF-8 and PMo_12_@ZIF-8-AC was further performed (Figure 6d). For PMo_12_@ZIF-8, PMo_12_ is mainly expressed by a sharp peak registered at −1.8 ppm, which may represent the isolated PMo_12_ incorporated into the MOF framework [46], and a slightly broad protuberance can also be observed at a higher chemical shift (around 7 ppm). This may be attributed to the interaction between PMo_12_ and the ZIF-8 framework [22]. The analysis of the catalyst after catalytic use (PMo_12_@ZIF-8-AC) presents the same sharp peak; however, the broad peak is now clearly observed around 7.4 ppm. This may indicate a higher interaction of the active center PMo_12_ with the ZIF-8 framework promoted by the oxidative catalytic reaction, i.e., further variations on the interaction between PMo_12_ and its environment linked to the framework’s transformation after catalytic use. This interaction may guarantee the superior catalytic activity of PMo_12_@ZIF-8 compared to the PMo_12_@ZIF-67, which probably promoted the alteration of ZIF-8 structure in consecutive ECODS cycles without the occurrence of PMo_12_ leaching (confirmed by ^31^P NMR analysis of the reaction solution medium at the end of catalytic experiments). On the other hand, the ability of the ZIF-67 to decompose H_2_O_2_ is well reported [47]; however, the quantification of H_2_O_2_ after PMo_12_@ZIF-67 catalytic use indicates the presence of at least half of the initial amount of the used oxidant. A similar H_2_O_2_ quantification after catalytic use was found for the ECODS system catalyzed by PW_12_@ZIF-8, indicating the absence of H_2_O_2_ consumption, which was probably caused by an absence of reactant diffusion into pore cavities and the non-occurrence of PW_12_ interaction. Therefore, the weakness (using PW_12_@ZIF-8) or absence (using PMo_12_@ZIF-67) of the catalytic activity of ZIF-based composites must be associated with a lower diffusion of reactants to their cavities, promoted by the higher POM loadings, or due to the absence of an interaction of the active POM with the MOF framework during the oxidative reaction.

## 4. Concluding Remarks

ZIF-based composites encapsulating phosphomolybdic acid (PMo_12_) and phosphotungstic acid (PW_12_) were successfully prepared, characterized, and further investigated as heterogeneous catalysts for extraction and catalytic oxidative desulfurization systems (ECODS): PMo_12_@ZIF-8, PMo_12_@ZIF-67, and PW_12_@ZIF-8. The catalytic efficiency of these composites was further compared with the pristine supports ZIF-8 and ZIF-67, and with the homogeneous active centers PMo_12_ and PW_12_. The supports revealed an absence of catalytic activity for the desulfurization of a multicomponent model diesel. The PMo_12_@ZIF-8 composite showed a much higher catalytic efficiency to oxidatively desulfurize various benzothiophene derivatives (BT, DBT, MDBT, and DMDBT) than the analogues PW_12_@ZIF-8. The most prominent difference between the composites is in fact their polyoxometalate loading abilities, since the homogeneous catalytic activity of PMo_12_ and PW_12_ is similar under the studied conditions. The PW_12_@ZIF-8 presents a higher loading (ten times) than the PMo_12_@ZIF-8. This high loading may promote a blockage of ZIF-8 cavities and a low diffusion of reactants, which results in a low catalytic activity of the composite. On the other hand, the PMo_12_@ZIF-67 did not present any catalytic activity, i.e., the incorporation of the highly active PMo_12_ into ZIF-67 resulted in an inactive material where the active center PMo_12_ cannot fulfill its role as the catalytic active center. In this last composite, a high loading of the guest compounds cannot be the reason for the absence of activity. The nature of the metallic center Co instead of Zn in the ZIF-8 framework, seems to play an important role in the catalytic performance of the catalyst. In fact, an interaction between the PMo_12_ and the ZIF-8 supporting structure was found, which is probably the reason for the high catalytic efficiency of the PMo_12_@ZIF-8 composite. This catalyst was recycled for nine consecutive desulfurization cycles, maintaining its activity without the occurrence of PMo_12_ leaching.

The work reported in the present manuscript corresponds to a “kick-off” study of this family of composite materials based on Keggin POMs incorporated into porous ZIFs (both ZIF-8 and ZIF-67) to be applied in the desulfurization of model fuels. In fact, the optimization of this type of material composition as well as their thermal modification is now being investigated to improve the catalytic oxidation performance of sulfur-containing compounds.

## Figures and Tables

**Table 1:** BET optimized parameters calculated for of both ZIF structures and POM@ZIF composite materials.

Sample	S_BET_ (m^2^/g)	V_P_ (cm^3^/g)
ZIF-8	1743	0.61
ZIF-67	1712	0.58
PMo_12_@ZIF-8	1044	0.37
PW_12_@ZIF-8	490	0.18
PMo_12_@ZIF-67	711	0.24

(S_BET_): BET specific surface area; (V_p_): total pore volume determined at P/P_o_ = 0.99.

**Table 2:** Comparison of catalytic efficiency of reported PMo_12_@MOFs composites for desulfurization of model fuels.

Catalyst	Sulfur	Temperature (°C)	Time (h)	Oxidant	Efficiency (%)	Reference
PMo_12_@NH_2_-MIL-101	1-BT, DBT, 4-MDBT, and 4,6-DMDBT	50	3	H_2_O_2_	95	[44]
PMo_12_@UiO-66 ^c^	DBT	60	1	TBHP	100	[38]
PMo_12_@UiO-66	DBT	60	1	H_2_O_2_	100	[39]
PMo_12_@MOF-199	4,6-DMDBT	120	1.5	O_2_	90	[44]
PMo_12_@UiO-67	T ^d^ and DBT	60	0.5	H_2_O_2_	100	[11]
PMo_12_@ZIF-67	DBT	70	3	TBHP	98	[40]
PMo_12_@MOF-808	1-BT, DBT, and 4-MDBT	70	1	H_2_O_2_	100	[45]
PMo_12_@MOF-808	DBT	50	1.5	H_2_O_2_	100	[41]
PMo_12_@ZIF-8-PIL ^a^	DBT	r.t. ^b^	2	H_2_O_2_	100	[42]
PMo_12_@DUT-67	DBT	50	1.5	H_2_O_2_	98	[43]
PMo_12_@ZIF-8	1-BT, DBT, 4-MDBT, and 4,6-DMDBT	70	2	H_2_O_2_	91	This work

^a^ PIL: porous ionic liquids. ^b^ r.t.: room temperature. ^c^ calcinated composite. ^d^ T: thiophene.

## Data Availability

Not applicable.

## Sample Availability

Samples of the compounds can be available by request from the authors.
